# Photosynthesis Mediated by *RBOH*-Dependent Signaling Is Essential for Cold Stress Memory

**DOI:** 10.3390/antiox11050969

**Published:** 2022-05-14

**Authors:** Qinghua Di, Yansu Li, Shuzhen Li, Aokun Shi, Mengdi Zhou, Huazhong Ren, Yan Yan, Chaoxing He, Jun Wang, Mintao Sun, Xianchang Yu

**Affiliations:** 1 Institute of Vegetables and Flowers, Chinese Academy of Agricultural Sciences, Beijing 100081, China; 82101189108@caas.cn (Q.D.); liyansu@caas.cn (Y.L.); 82101196061@caas.cn (A.S.); 82101199108@caas.cn (M.Z.); yanyan@caas.cn (Y.Y.); hechaoxing@caas.cn (C.H.); wangjun01@caas.cn (J.W.); 2 Engineering Research Center of the Ministry of Education for Horticultural Crops Breeding and Propagation, College of Horticulture, China Agricultural University, Beijing 100193, China; renhuazhong@cau.edu.cn; 3 Beijing Key Laboratory of Growth and Developmental Regulation for Protected Vegetable Crops, College of Horticulture, China Agricultural University, Beijing 100193, China; 4 College of Life Science, Gannan Normal University, Ganzhou 341000, China; 1600082@gnnu.edu.cn

**Keywords:** brassinosteroids, maintenance of acquired cold tolerance, photosynthetic efficiency, respiratory burst oxidase homologue

## Abstract

Cold tolerance is improved by cold stress acclimation (CS-ACC), and the cold tolerance level is ‘remembered’ by plants. However, the underlying signaling mechanisms remain largely unknown. Here, the CS memory mechanism was studied by bioinformation, plant physiological and photosynthetic parameters, and gene expression. We found that CS-ACC induced the acquisition of CS memory and enhanced the maintenance of acquired cold tolerance (MACT) in cucumber seedlings. The H_2_O_2_ content and NADPH oxidase activity encoded by *CsRBOH* was maintained at higher levels during recovery after CS-ACC and inhibition of *RBOH*-dependent signaling after CS-ACC resulted in a decrease in the H_2_O_2_ content, NADPH oxidase activity, and MACT. *CsRBOH2*, *3*, *4*, and *5* showed high expression during recovery after CS-ACC. Many BZR-binding sites were identified in memory-responsive *CsRBOHs* promoters, and *CsBZR1* and *3* showed high expression during recovery after CS-ACC. Inhibition of *RBOH*-dependent signaling or brassinosteroids affected the maintenance of the expression of these memory-responsive *CsRBOHs* and *CsBZRs.* The photosynthetic efficiency (PE) decreased but then increased with the prolonged recovery after CS-ACC, and was higher than the control at 48 h of recovery; however, inhibition of *RBOH*-dependent signaling resulted in a lower PE. Further etiolated seedlings experiments showed that a photosynthetic capacity was necessary for CS memory. Therefore, photosynthesis mediated by *RBOH*-dependent signaling is essential for CS memory.

## 1. Introduction

Environmental temperatures are always fluctuating, some of which is regular while other change is irregular and exhibits aperiodicity. The stress caused by fluctuations in the environmental temperature on plants can be transient or persistent [1,2]. Stress acclimation can enhance the tolerance of plants, which can be maintained under non-stress conditions. Certainly, once plants have been in a normal environment for a longer time, they forget this tolerance to maximize their recovery for growth and development [1,3,4]. Temperature is an important environmental factor that affects plant growth, development, and production and restricts the geographical distribution of plants [5,6,7,8,9]. Plants, as sessile organisms, can temporally perceive and adapt to changing environmental temperatures [10,11]. Plant temperature stress tolerance can be divided into three types: basic tolerance (BT), acquired tolerance (AT), and maintenance of acquired tolerance (MAT). BT refers to temperature tolerance without any stress adaptation. However, plants can acquire tolerance from moderate-temperature stress, which allows it to survive severe-temperature stress that would otherwise be lethal to non-adapted plants [12]. AT can be maintained over several days (also called short temperature stress memory) regardless of whether the plant is exposed to temperature stress after temperature stress acclimation [4], which is called MAT. It is an active process that can be genetically separated from BT and AT [1,13,14,15,16].

When plants suffer cold stress, plant cells show increased intracellular Ca^2+^, which is activated by cyclic nucleotide-gated ion channels (CNGCs) [17,18]. Calcium-dependent protein kinases (CPKs) sense changes in cytoplasmic Ca^2+^ levels [19,20,21] and interact with downstream signaling molecules, including hormones [22,23], mitogen-activated protein kinases (MPKs) [24,25], and reactive oxygen species (ROS) [8,26], leading to adaptation to cold stress. At present, research investigating cold mechanisms has mainly focused on BT or AT while studies on MAT are limited [27]. In response to cold stress, plants maintain their cold stress tolerance after cold stress acclimation (CS-ACC) during recovery [3]. Currently, studies on the maintenance of acquired cold tolerance (MACT) have only been reported in *Arabidopsis* and were limited to the mining of transcriptome and metabolome datasets [13]. Moreover, studies have shown that MAT is regulated by both intracellular signaling and epigenetic modifications [1]. However, the underlying signaling mechanism that regulates MACT in plants remains largely unclear [27]. When plants are subjected to recurrent cold stress, what specific signaling pathways are required during recovery after CS-ACC to regulate MACT? Because cold stress is a recurrent environmental stimulus that occurs during the process of plants’ natural growth, this type of cold stress is typical of natural environments. Therefore, studying the mechanism of MACT not only helps to improve plant cold tolerance but also has important theoretical significance and scientific value to enrich the mechanism of plant resistance to abiotic stress in natural environments.

Hydrogen peroxide (H_2_O_2_) produced by the NADPH oxidase (NADPH-H_2_O_2_) encoded by *respiratory burst oxidase homologue* (*RBOH*) is a signaling factor in many abiotic stresses and enhances plant tolerance [26,28,29,30,31,32]. Our previous studies have shown that *RBOH*-dependent signaling is involved in regulating heat stress memory in tomato [33,34]. However, whether *RBOH*-dependent signaling also participates in cold stress memory regulation in plants and the regulation mechanism involved remains unknown.

Plants also responded cold stress by activating hormone signaling pathways. Ethylene insensitive 3 (EIN3) is a key transcription factor in ethylene signaling and negatively regulates cold tolerance in *Arabidopsis* by binding to the CBFs promoter [35]. Cold stress also prevents jasmonic acid from inhibiting ICE1/2 by JAZ1/4, the repressor of the jasmonic acid signaling pathway ZIM domain protein, and positively regulates *ICE* transcription and *CBF1-3* expression [36]. Mutations of Brassinosteroid-insensitive factor 2 (*BIN2*) and its homologous genes enhance the freezing resistance of plants [37]. However, three transcription factors downstream of BIN2, BZR1 (Brassinazole-resistant 1), BES1 (Bin1-EMS-supressor 1), and CESTA positively regulate the expression of *CBFs* and plants’ cold tolerance [37,38], suggesting that Brassinosteroids (BRs) signaling is complex response that occurs during cold stress, and might play a more important role in the cold tolerance of plants.

As a class of steroid hormones, BRs are perceived by the leucine-rich repetitive receptor kinases (LRR RLKs) located on the plasma membrane [39]. Studies have shown that in addition to their indispensable role in the growth and development of plants, BRs are also implicated in plants’ tolerance of various stresses [40]. BR signaling is mainly transmitted through the plasma membrane receptor BRI1, which influences the phosphorylation/dephosphorylation of *BES1*/*BZR1* and subsequent binding to promoters of genes that regulate growth and development, and biological and abiotic stress, thus influencing the regulation of plant growth and stress resistance [41,42]. Studies have shown that the stress tolerance activated by BR signaling is related to the increase in H_2_O_2_ produced by the activity of NADPH oxidase encoded by *RBOH* [40,43]. However, it is also unclear whether BRs participate in MACT regulation, and the underlying mechanism is still unknown.

Photosynthesis is an important performance parameter in agriculture and the recovery of photosynthetic performance is crucial to plant growth and production [44,45,46]. Plants’ photosystem integrity and CO_2_ assimilation are significantly affected by low-temperature stress [47,48,49,50]. Indicators for evaluating plant photosynthesis, including the variation in chlorophyll fluorescence parameters, have been widely used in the study of plant environmental stress, especially for F_V_/F_M_ and PI_ABS_ [51,52,53]. However, how photosynthesis responds to CS-ACC events and whether *RBOH*-dependent signaling regulates photosynthesis during recovery after CS-ACC are still unclear.

Cucumber (*Cucumis sativus* L.) is a globally important vegetable in the *Cucurbitaceae* family. It is a typical cold-sensitive vegetable with important economic and nutritional value [54]. In recent years, cucumber has achieved continuous annual production, and the planting area for cultivation has expanded. However, cold stress significantly restricts its quality and yield, which has become a key environmental challenge and limiting factor affecting the growth and development of this crop plant [55]. Here, we found that CS-ACC resulted in the acquisition of cold stress memory and enhancement of MACT cucumber seedlings. *RBOH*-dependent signaling and BRs were essential for the cold stress memory of cucumber seedlings and *RBOH*-dependent signaling was essential for MACT by altering the photosynthetic efficiency during recovery after CS-ACC.

## 2. Material and Methods

### 2.1. Plant Materials and Temperature Treatments

The cultivated cucumber variety ‘Changchun dense thorn’ (inbred line of the research group) was used as the experimental material. Selected cucumber seeds with full grains and of the same size were wrapped in clean gauze and soaked in 50 °C water for 30 min and incubated in dark conditions at 28 °C to promote germination. Cucumber seeds with consistent germination were randomly selected and sown in a square (7 × 7 cm) seedling pot filled with substrate (peat: vermiculite = 2:1, *V*:*V*). The seedlings were raised in a greenhouse (No. 10) at the Institute of Vegetables and Flowers at the Chinese Academy of Agricultural Sciences. The seedlings were moved to an artificial climate chamber after about 20 days (i.e., 2 true leaf stage) for pre-culture for about 5 days, and then treated. Seedlings were cultured at a temperature of 25 °C/18 °C (day/night) with a photoperiod of 12 h light/12 h dark (day/night), at a light intensity (PPFD) of 250 ± 10 μmol·m^−2^·s^−1^. The relative humidity was 60–80% and 1/2 Hoagland nutrient solution was irrigated once during the growth period. Since diphenyleneiodonium (DPI) and brassinozole (BRZ) were dissolved in ethanol, the control was also sprayed with the corresponding concentration of ethanol during recovery after CS-ACC. This experiment included the following six treatments:

(1) CK: 25 °C.
(2) Cold stress induction (CS-I) combined with a short recovery: 10 (1 h) and 25 °C (1.5 h).
(3) Cold stress induction (CS-I) combined with a long recovery: 10 (1 h) and 25 °C (48 h).
(4) Cold stress acclimation (CS-ACC) combined with a long recovery: 10 (1 h), 25 (1.5 h), 1 (3 h), and 25 °C (48 h).
(5) DPI was sprayed on the seedlings after CS-ACC (CS-ACC-DPI) combined with a long recovery: 10 (1 h), 25 (1.5 h), and 1 °C (3 h), 50 mmol·L^−1^ DPI, 25 °C (48 h).
(6) BRZ was sprayed onto the seedlings after CS-ACC (CS-ACC-BRZ) combined with a long recovery: 10 (1 h), 25 (1.5 h), and 1 °C (3 h), 50 mmol·L^−1^ BRZ, 25 °C (48 h).

After CS-ACC, cucumber seedlings were sprayed with DPI or BRZ, respectively, and the leaves were collected at 4, 24, and 48 h during recovery (including CK). The leaves were immediately placed in liquid nitrogen and stored at −80 °C for gene expression analysis and assessment of the physiological parameters. The fluorescence parameters were determined in vivo. After treatment, the seedlings were exposed to 1 °C for 20–24 h and then placed in recovery for about 7 days at a temperature of 25 °C/18 °C (day/night), and the chilling injury index (CII) for each group was measured as described by Zhang et al. [56] and Liu et al. [57] with minor modifications (1):

(1) $$\mathrm{CII}=\frac{\sum \mathrm{Number}\;\mathrm{of}\;\mathrm{Plants}\;\mathrm{of}\;\mathrm{per}\;\mathrm{Grade}\times \mathrm{Correspond}\;\mathrm{ing}\;\mathrm{Grade}}{\mathrm{Number}\;\mathrm{of}\;\mathrm{Total}\;\mathrm{Plants}\times \mathrm{Highest}\;\mathrm{Grade}}\times 100\%$$

The chilling injury levels of individual plants were investigated according to the following seedling leaf classification standards:

Grade 0: cotyledons and true leaves were intact without obvious injury symptoms.
Grade 1: green cotyledons were wilted and the leaf margins on true leaves were wilted and curly.
Grade 2: dehydration spots were apparent on the true leaves but were less than 1/2 of the total leaf area.
Grade 3: the area of dehydration spots on the true leaves was 1/2 of the leaf area and the heart leaf margin was damaged.
Grade 4: the area of dehydration spots on true and heart leaves was more than 1/2 of the leaf area.
Grade 5: the whole plant was dehydrated and wilted.

### 2.2. Genome-Wide Identification of the RBOH and BZR Families

The latest version of the cucumber genome website (V3.0; http://cucurbitgenomics.org/organism/20, accessed on 29 December 2021) was used to obtain the gene ID, strands, location, length, chromosome, and other family members. We first downloaded the reported amino acid sequences of 10 *RBOH* family members from an *Arabidopsis* database (http://www.arabidopsis.org, accessed on 9 April 2015) and searched for the cucumber *CsRBOH* genes using blastp homology. The Pfam (http://pfam.xfam.org/, accessed on 19 November 2021) was used to identify the conserved domain of similar protein sequences by setting the default parameters. A hidden Markov model of cucumber *RBOH*-specific family domains was established with a hmmsearch (http://hmmer.org/, accessed on 25 July 2020). Using the SMART online (http://smart.embl-heidelberg.de/, accessed on 26 October 2020) and NCBI CDD (https://www.ncbi.nlm.nih.gov/cdd, accessed on 22 February 2021) to determine the integrity of conserved domains and filter redundant sequences, we identified the *CsRBOH* family members. This method was also used to identify the cucumber *CsBZR* family members. We predicted the subcellular location of plant proteins using online resources (http://www.csbio.sjtu.edu.cn/bioinf/Cell-PLoc-2/, accessed on 12 December 2010). The distribution of gene positions on chromosomes was mapped using the software Mapchart.

### 2.3. Phylogenetic Tree, Gene Structure, and Conserved Domains in RBOH and BZR Families

The phylogenetic relationships between the RBOH and BZR family were investigated, respectively. The sequences of different plants, including tomato, rice, and tobacco, were obtained from UniProt. There were 40 *RBOH* in total, including 10 in *Arabidopsis*, 5 in tomato, 7 in rice, 9 in tobacco, and 9 in cucumber, while there were 29 *BZR*, including 6 in *Arabidopsis*, 7 in tomato, 4 in rice, 8 in tobacco, and 4 in cucumber. Multi-sequence similarity comparison was performed for the *RBOH* and *BZR* family in the five species using Clustal Omega. Phylogenetic trees were constructed with MEGA7.0 using the neighbor joining method. The default parameters of the bootstrap values were used as verification parameters and set to 1000, with the ‘Poisson Model’ used to verify the reliability.

Multiple sequences of conserved motifs in the RBOH and BZR family were analyzed online using MEME. The conserved domains were obtained from SMART and NCBI CDD. According to the GFF file provided by the cucumber genome database, the location of exons, CDS, and UTR of these genes on the chromosomes were obtained and used for GSDS to construct gene structural diagrams.

MCScanX was used to analyze the tandem and fragment replication of the cucumber *CsRBOH* and *CsBRZ* family. The homology of the *RBOH* and *BRZ* family members between cucumber and other plant species was studied by comparative genomics to explore the evolution mechanism of the two gene families.

### 2.4. Cis-Acting Element Analysis

The analysis of the cis-acting element was performed by a Perl script. We first downloaded the sequences of the *RBOH* family members from the cucumber V3.0 version genome database, and then intercepted the DNA sequence of the promoter region (1500 bp upstream of the transcription start site) with the Perl script, and searched for cis regulatory elements (activation site and inhibition site) recognized by *BZR* throughout the DNA sequence of the promoter region.

### 2.5. RNA Extraction and qRT-PCR Analysis

Total RNA was collected from cucumber samples using a plant-specific polysaccharide polyphenol total RNA Extraction Kit (DP441, Tiangen Biotech Co., Ltd., Beijing, China). RNA integrity was detected using 2 μL of extracted RNA for 1% agarose gel electrophoresis while the RNA concentration was measured with a Biodrop (BioLion Technology Co., Ltd., Cambridge, UK) spectrophotometer. First-strand cDNA was synthesized by reverse transcription according to the instructions for the PrimeScript™ RT reagent Kit with gDNA Eraser (RR047A, Takara Biomedical Technology Co., Ltd., Beijing, China). The synthesized first-strand cDNA was diluted 10 times and used as a template for quantitative real-time PCR (qRT-PCR). qRT-PCR was performed on an Mx3000P real-time quantitative fluorescent PCR machine (Agilent Technologies, Inc., Santa Clara, CA, USA) following the instructions for the SYBR^®^ Premix Ex Taq™ Kit (RR420A, Takara Biomedical Technology Co., Ltd., Beijing, China). The second leaf from the top from five cucumber seedlings was considered a biological replicate, and each treatment included three replicates. The relative gene expression was calculated using the 2^−^^ΔΔCt^ method [58]. Each primer of *CsRBOHs* and *CsBZRs* is presented in Table S1.

### 2.6. Chlorophyll Fluorescence-Induced Kinetic Curve (OJIP) Measurement

The determination of the rapid chlorophyll (chl) fluorescence-induced kinetics curve and the calculation of relevant fluorescence parameters were conducted according to the methods of Turan et al. [47] and Di et al. [59]. After CS-ACC, cucumber seedling leaves were placed in darkness with a small clip for at least 30 min and then the continuous excitation fluorescence was measured with a Plant Efficiency Analyzer (Handy PEA, Hansatech, UK) [60]. Plants were measured with 3000 μmol·m^−2^·s^−1^ pulsed red light, and the fluorescence signal was recorded from 10 μs to 1 s at an initial recording speed of 100,000 times per second. The OJIP curve was analyzed using the JIP test as previously described by Turan et al. [47] and Masojídek et al. [61].

### 2.7. Measurement of the Electrolytic Leakage; Contents of Proline, H_2_O_2_, and Chlorophyll; and NADPH Oxidase Activity

Measurement of the electrolytic leakage was conducted according to Fang et al. [62]. An FE30 conductivity meter (Mettler Toledo International Co., Ltd., Zurich, Switzerland) was used to measure the distilled water (EC3) and conductivity of 0.3 g of cucumber seedling leaves placed in 30 mL of distilled water before boiling (EC1) and after boiling (EC2), respectively. The following calculation formula was used:

(2) $$\mathrm{Electrical}\;\mathrm{leakage}=\frac{\left(\mathrm{EC}1-\mathrm{EC}3\right)}{\left(\mathrm{EC}2-\mathrm{EC}3\right)}\times 100\%$$

A Comin kit (Suzhou Comin Biotechnology Co., Ltd., Suzhou, China) was used to determine the proline and H_2_O_2_ content. In total, 0.1 g of fresh cucumber seedling leaves were collected and the extract was added. Then, the samples were ground and centrifuged to obtain the crude enzyme extract for analysis according to the instructions of the kit. Measurement of the proline content was carried out using the sulfosalicylic acid assay at OD520 nm [63]. The H_2_O_2_ content was determined by the TiCl_4_ precipitation assay at OD415 nm [64]. The NADPH oxidase activity was determined using an ELISA kit (Jiangsu MEIMIAN Co., Ltd., Yancheng, China) according to the manufacturer’s instructions. Detection of the chlorophyll content was conducted as described by Arnon [65] with minor modifications. The contents of chlorophyll a (Ca), chlorophyll b (Cb), and carotenoid (Cc) were calculated as follows:

(3) Ca=13.95×OD665−6.88×OD649

(4) Cb=24.96×OD649−7.32×OD665

(5) $$\mathrm{Cc}=\frac{1000\times \mathrm{OD}470-2.05\times \mathrm{Ca}-114.8\times \mathrm{Cb}}{245}$$

### 2.8. Statistical Analysis

SPSS 18.0 (SPSS, Inc., Chicago, IL, USA) was used to analyze the significance of difference in Turkey HSD (*p* < 0.05) and Student’s *t* test (*p* <0.05). The data were repeated ≥3 times.

## 3. Results

### 3.1. Cold Stress Induced the Acquisition of Cold Stress Memory in Cucumber Seedlings and Enhanced the Acquired Cold Tolerance

**Figure 1:**
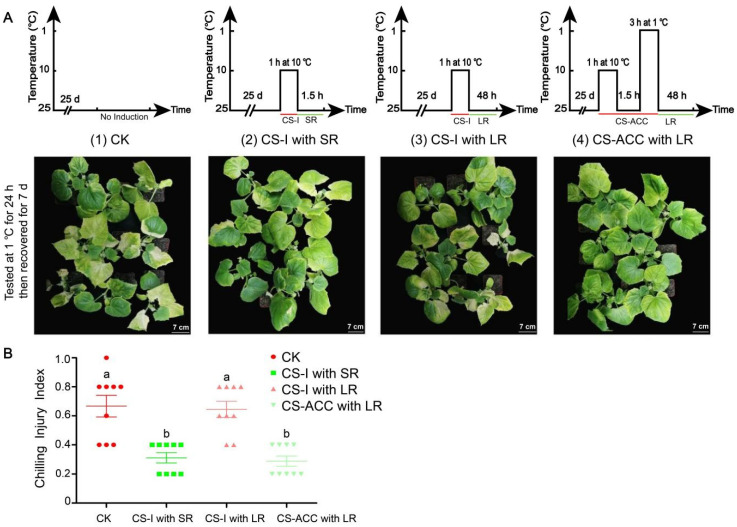
The cold tolerance (**A**) and chilling injury index (CII) (**B**) of cucumber seedlings tested by a tester cold stress (1 °C, 24 h) after different treatments, and then placed in recovery for a period (25 °C/18 °C, day/night, 7 days). (**A**): a schematic diagram and cold tolerance of different treatments of cucumber seedlings. CK: cucumber seedlings with no acclimation and at 25 °C during treatment; CS-I with SR: cucumber seedlings were treated with a cold stress regime of 10 °C for 1 h and 25 °C for 1.5 h; CS-I with LR: cucumber seedlings were treated with a cold stress regime of 10 °C for 1 h and 25 °C for 48 h; CS-ACC with LR: cucumber seedlings were treated with a cold stress regime of 10 °C for 1 h, 25 °C for 1.5 h, 1 °C for 3 h, and 25 °C for 48 h; CS-I: cold stress induction; CS-ACC: cold stress acclimation; SR: short recovery; LR: long recovery. (**B**): the chilling injury index (CII) of cucumber seedlings. The different letters indicate significant differences as assessed by the Turkey HSD test (*p* < 0.05; *n* = 9).

A tester cold stress (1 °C) was applied for 24 h to determine the cold tolerance of the treatments. The cold tolerance was significantly enhanced following a 1.5 h recovery after induction at 10 °C (Figure 1A). In contrast, the cold tolerance was significantly decreased following a 48 h recovery period after induction at 10 °C (Figure 1A). However, the cucumber seedlings showed stronger cold tolerance following a 48 h recovery after cold stress acclimation (CS-ACC) that included the following regime: 1 h at 10 °C, 1.5 h at 25 °C, and 3 h at 1 °C (Figure 1A). These results were further confirmed by the chilling injury index (CII) (Figure 1B). The above results indicated that CS-ACC resulted in the acquisition of cold stress memory in cucumber seedlings and enhanced the maintenance of acquired cold tolerance (MACT).

### 3.2. Genome-Wide Analysis of the RBOH Protein Family in Cucumber

To understand the presentation of the *RBOH* family in cucumber, we performed a genome-wide analysis of the RBOH protein family. We first identified nine *CsRBOH* genes of the cucumber genome through hmmsearch. The specific locations of these members were found to be distributed on chromosome 1, 3, 4, 5, and 6 (Figure S1). According to the order of the *CsRBOH* genes on the cucumber chromosomes, these *CsRBOH* genes were named *CsRBOH1*-*9*, respectively. Subcellular localization prediction showed that all the family members were localized on cell membranes (Table S2).

We further constructed protein phylogenetic trees of different plants for the RBOH family (Figure S2A). The RBOH rootless phylogenetic tree was divided into six groups. Groups 1, 2, and 4 contained 34 RBOH proteins, accounting for 90% of the family; group 3 and 5 contained 1 RBOH protein, respectively; and group 6 contained 2 RBOH proteins, including 1 protein in cucumber (Figure S2A). The branch density of groups 1, 2, and 4 was higher than that of groups 3, 5, and 6, indicating that the evolution degree of RBOH proteins in groups 1, 2, and 4 was higher than that of the other groups. However, the RBOH proteins in groups 3, 5, and 6 may be more easily lost during evolution. Eight of the nine RBOH proteins in cucumber were more highly evolved except for *CsRBOH1*, which was relatively more conserved across the phylogeny. In addition, we searched for the RBOH proteins through the MEME online suite and identified 10 conserved motifs (Figures S2B and S3). Members of the same group retained similar motifs, in which motifs 2, 4, 5, 8, and 9 appeared in each gene while motifs 1 and 2 were highly conserved amino acid residues in the RBOH domain. Motifs 8 and 10 were missing in *CsRBOH1*, *5*, and *8* in cucumber, respectively. We also found that all *RBOH* genes in this study contained UTR, intron, and CDS regions of different lengths (Figure S2C). These results indicated that the RBOH family in cucumber is mainly distributed in the groups with more members in the phylogenetic tree of four model species; therefore, the RBOH family is relatively conserved in the protein sequences.

### 3.3. Collinearity Analysis of the RBOH Family in Different Plant Species

To further understand the gene linkage, type, and relative sequence conservation of *RBOH* in different plant species, we used comparative genomics to investigate the collinearity of the *RBOH* family between cucumber and different plant species, including *Arabidopsis*, tomato, rice, and tobacco, and to evaluate the mechanism of *RBOH* evolution. We found that only two *CsRBOH* genes (*CsRBOH5* and *CsRBOH6*), which were located on chromosomes 4 and 5, respectively, had collinearity (Figure S4C). These two genes had highly similar motif domains and coding regions (Figure S2A). It is possible that genome replication facilitated the expansion of these gene families throughout plant evolution. By comparing and analyzing the *RBOH* family between cucumber and different plant species, only three of the nine *CsRBOH* genes had high homology with *Arabidopsis* (e.g., *CsRBOH3* and *AT1g09090*; *CsRBOH5* and *AT1g64060*; *CsRBOH6* and *AT1g64060*; Figure S4A). Additionally, three *CsRBOH* genes that had high homology with tomato were *CsRBOH3* and *Solyc01g099620*, *CsRBOH6* and *Solyc08g081690*, and *CsRBOH9* and *Solyc06g075570* (Figure S4D) while no *CsRBOHs* were found to share homology with rice (Figure S4B) or tobacco (Figure S4E). These results showed that *CsRBOH* was specific and highly evolved regarding its nucleotide sequence.

In general, the RBOH protein sequence of different species was conserved; however, the collinearity of *RBOH* sequences between cucumber and other species was relatively weak. Theis indicated that the nucleotide variation of *CsRBOH* did not affect the function of the RBOH protein.

### 3.4. A Large Number of BZR-Binding Sites Exist in the Promoter of the RBOH Family

**Figure 2:**
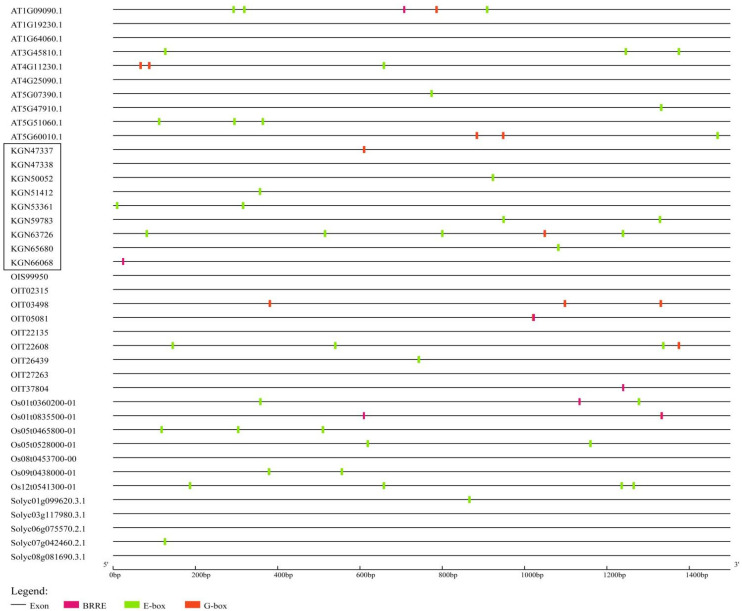
Analysis of BZR-binding sites in the 1500-bp DNA sequences upstream of the promoter of *RBOH* in different plant species (*Arabidopsis*, cucumber, rice, tobacco, and tomato). The black line represents the exons, the two red modules represent different inhibition sites (BRRE and G-box), and the green module represents the activation sites (E-box).

To analyze the sequences upstream of the *RBOH* family, the 1500-bp DNA sequences upstream of the transcription start site of *RBOH* in different plant species were intercepted through Perl script. A large number of BZR-binding sites were identified (Figure 2), including E-box activation sites and BRRE and G-box inhibition sites [37,66,67,68]. The analysis showed that 4 out of 5 plant species contained more than twice the number of active sites as inhibition sites, accounting for 80% of the plant species tested. Cucumber contained 11 active sites and 3 inhibition sites, *Arabidopsis* contained 13 active sites and 6 inhibition sites, rice contained 13 active sites and 3 inhibition sites, tomato contained only 2 active sites and no inhibition sites, and tobacco contained 4 active sites and 6 inhibition sites. Therefore, we hypothesized that the BZR transcription factor of BRs can widely bind to the promoter of the *RBOH* family.

### 3.5. Inhibition of BRs or RBOH-Dependent Signaling Affected the Maintenance of the Expression of Four CsRBOHs during Recovery after CS-ACC

**Figure 3:**
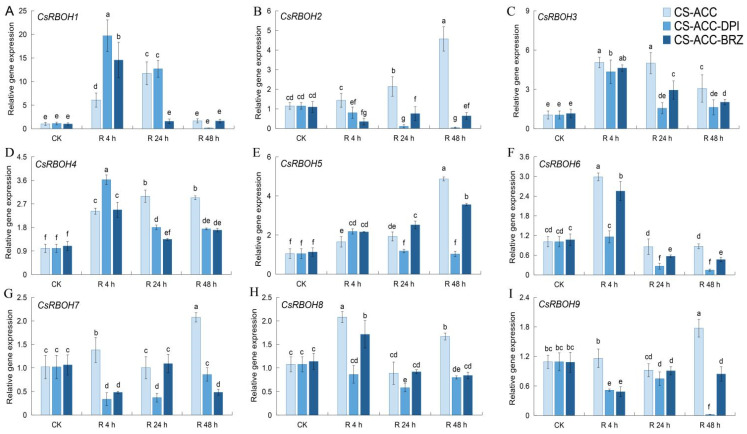
Inhibition of *RBOH*-dependent signaling or BRs during recovery after CS-ACC affected the expression of *CsRBOH1* (**A**), *CsRBOH2* (**B**), *CsRBOH3* (**C**), *CsRBOH4* (**D**), *CsRBOH5* (**E**), *CsRBOH6* (**F**), *CsRBOH7* (**G**), *CsRBOH8* (**H**) and *CsRBOH9* (**I**), respectively. CK: control, cucumber seedlings with no acclimation and at 25 °C during recovery; CS-ACC: cucumber seedlings were treated with a cold stress regime of 10 °C for 1 h, 25 °C for 1.5 h, and 1 °C for 3 h; CS-ACC-DPI: diphenyleneiodonium (DPI) was sprayed onto the seedlings during recovery after CS-ACC; CS-ACC-BRZ: brassinozole (BRZ) was sprayed onto the seedlings during recovery after CS-ACC; R: recovery, 25 °C. The different letters indicate significant differences as assessed by Turkey HSD test (*p* < 0.05; *n* ≥ 4).

We further determined the expression pattern of the *CsRBOH* family during recovery after CS-ACC. *CsRBOH2*, *3*, *4*, and *5* showed high expression during recovery after CS-ACC (Figure 3B–E). We called these genes the cold stress memory-responsive (CSM) genes. However, most of the other genes increased and then decreased during recovery after CS-ACC. When DPI or BRZ were sprayed, *CsRBOH*-CSM decreased during 24 and 48 h of recovery after CS-ACC (Figure 3) except for *CsRBOH5* after 24 h of recovery of CS-ACC-BRZ.

### 3.6. NADPH Oxidase Encoded by CsRBOHs Showed High Levels during Recovery after CS-ACC and Is Essential for Cold Stress Memory

**Figure 4:**
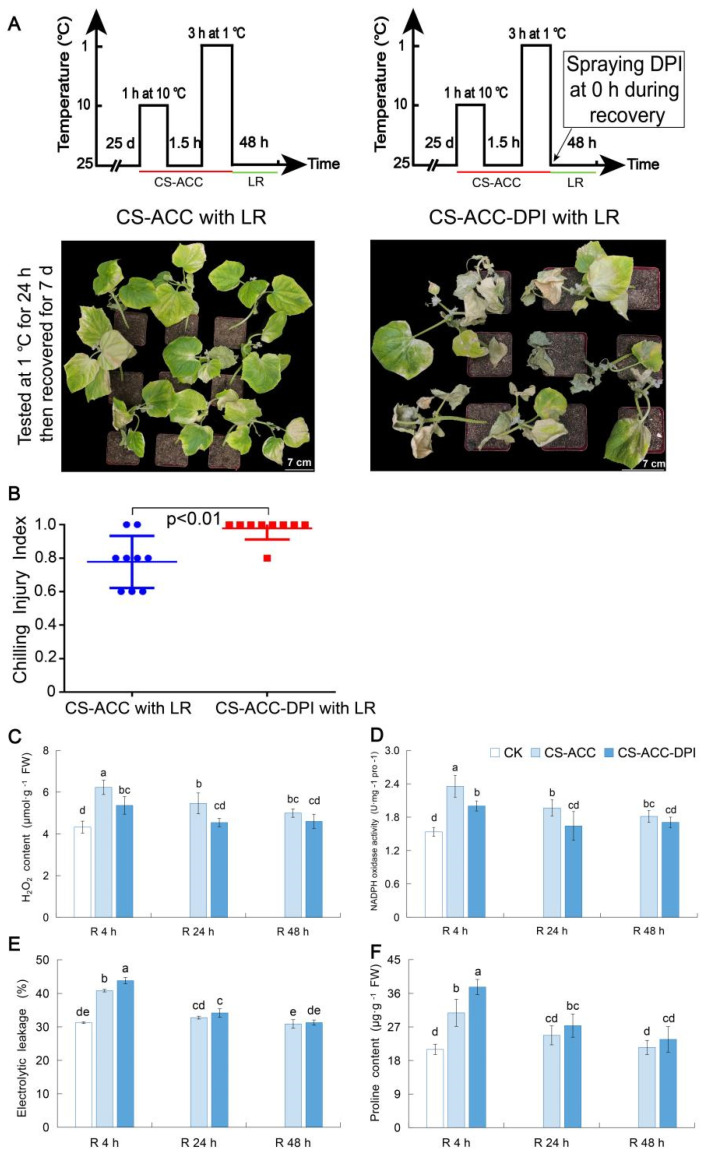
The cold tolerance (**A**) and CII (**B**) of cucumber seedlings tested by a tester cold stress (1 °C, 24 h) following 48 h of recovery after CS-ACC or CS-ACC-DPI, and then placed in recovery for a period (25 °C/18 °C, day/night, 7 days). (**A**): a schematic diagram and cold tolerance of CS-ACC with LR and CS-ACC-DPI with LR. CS-ACC: cucumber seedlings were treated with a cold stress regime of 10 °C for 1 h, 25 °C for 1.5 h, and 1 °C for 3 h; CS-ACC-DPI: DPI was sprayed onto the seedlings during recovery after CS-ACC. (**B**): CII of cucumber seedlings. (**C**–**F**): the H_2_O_2_ content, NADPH oxidase activity, electrolytic leakage, and proline content during recovery after CS-ACC or CS-ACC-DPI, respectively. CS-ACC: cold stress acclimation; LR: long recovery, 25 °C. The different letters indicate significant differences as assessed by the Turkey HSD test (*p* < 0.05; *n* ≥ 3).

In the *CsRBOH* family, we found that *CsRBOH*-CSM genes showed high expression during recovery after CS-ACC. Therefore, we hypothesized that *CsRBOH*-dependent signaling participates in cold stress memory. In this study, the cold tolerance and CII of cucumber seedlings were tested by a tester cold stress (1 °C) following 48 h of recovery after CS-ACC or CS-ACC-DPI. When the tester time reached 24 h, we found that after CS-ACC, CII was significantly lower than that of CS-ACC-DPI (Figure 4A,B). Thus, inhibition of *RBOH*-dependent signaling significantly reduced the cold tolerance of cucumber seedlings. Furthermore, we detected the total H_2_O_2_ content and the activity of NADPH oxidase encoded by the *CsRBOH* family. Compared with CK, the total H_2_O_2_ content and NADPH oxidase activity during recovery after CS-ACC significantly increased and was maintained at higher levels. After spraying DPI, the NADPH oxidase activity decreased, which resulted in a decrease in *RBOH*-dependent H_2_O_2_ signaling so that the total H_2_O_2_ content decreased during recovery after CS-ACC (Figure 4C,D). Meanwhile, inhibition of *RBOH*-dependent signaling increased the electrolytic leakage and proline content during 4 h of recovery after CS-ACC (Figure 4E,F). These results indicated that *RBOH*-dependent signaling was essential for the cold stress memory of cucumber seedlings.

### 3.7. Photosynthesis Efficiency Mediated by CsRBOH-Dependent Signaling Was Essential for Cold Stress Memory

**Figure 5:**
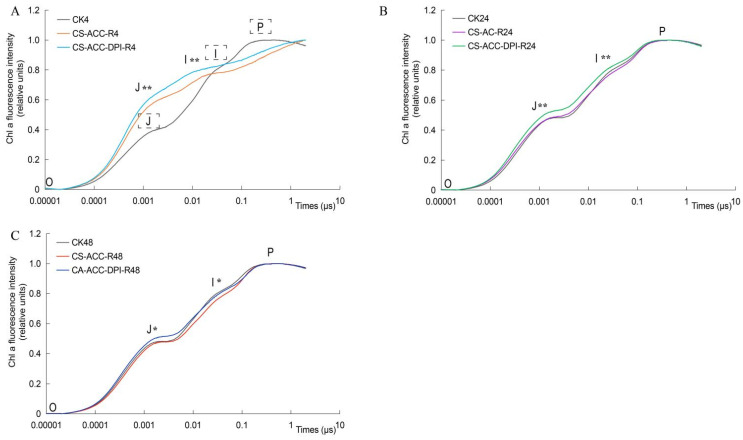
*RBOH*-dependent signaling affected the photosynthetic efficiency of PSII during recovery after CS-ACC in cucumber seedlings. (**A**–**C**): the changes in the Chl a fluorescence induction curve during 4 (**A**), 24 (**B**), and 48 h (**C**) of recovery, respectively, after CK, CS-ACC, and CS-ACC-DPI treatment, respectively. CK: the control, cucumber seedlings with no acclimation and at 25 °C during recovery; CS-ACC: cucumber seedlings were treated with a cold stress regime of 10 °C for 1 h, 25 °C for 1.5 h, and 1 °C for 3 h; CS-ACC-DPI: DPI was sprayed onto the seedlings during recovery after CS-ACC. CS-ACC: cold stress acclimation; R: recovery, 25 °C. Point O: initial fluorescence intensity when the PSII reaction center was completely open; Point J: fluorescence intensity when electron receptors Q_A_ were in an initial instantaneous maximum reduction state, which reflects the reduction rate of Q_A_; Point I: the size of the reduced plastiquinone (PQ) library; Point P: fluorescence intensity of all electron receptors in PSII that are in the maximum reduced state. Different colored lines represent different treatments. Significant differences for A and C are indicated for the Tukey HSD test (3 treatments; *p* < 0.05; *n* ≥ 4). Significant differences for B are indicated for Student’s *t* test (2 treatments; *p* < 0.05; *n* ≥ 4). “*” represents the 0.05 level, and “**” represents the 0.01 level.

To explore whether *RBOH*-dependent signaling affected the photosynthesis efficiency (PE) during recovery after CS-ACC, we determined the PE-related fluorescence induction curves (Figure 5). Compared with CK, the fluorescence intensity (FI) for the I and J points of seedlings during recovery after CS-ACC gradually decreased and FI was even lower after 48 h of recovery than CK, indicating that PE gradually increased during recovery after CS-ACC. However, inhibition of *RBOH*-dependent signaling resulted in higher FI (Figure 5), indicating that PE of CS-ACC-DPI was suppressed.

**Figure 6:**
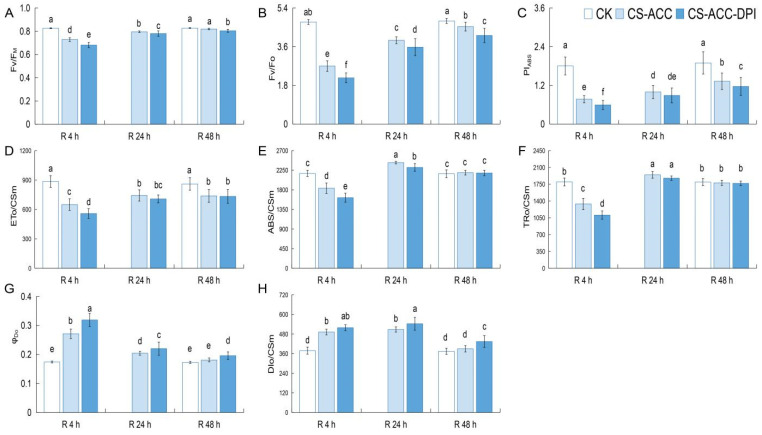
*RBOH*-dependent signaling affected the different photosynthetic efficiency-related parameters F_V_/F_M_ (**A**), F_V_/F_O_ (**B**), PI_ABS_ (**C**), ETo/CSm (**D**), ABS/CSm (**E**), TRo/CSm (**F**), φ_Do_ (**G**), DIo/CSm (**H**) of PSII during recovery after CS-ACC in cucumber seedlings. CK: control, cucumber seedlings with no acclimation and at 25 °C during recovery; CS-ACC: cucumber seedlings were treated with a cold stress regime of 10 °C for 1 h, 25 °C for 1.5 h, and 1 °C for 3 h; CS-ACC-DPI: DPI was sprayed onto the seedlings during recovery after CS-ACC. CS-ACC: cold stress acclimation; R: recovery, 25 °C. F_V_/F_M_: maximum light energy conversion efficiency of the PSII reaction center; F_V_/F_O_: the potential photochemical activity of PSII; PI_ABS_: performance index based on the absorbed light energy; ETo/CSm: the energy captured by per unit leaf section for electron transport; ABS/CSm: the light energy absorbed per unit leaf section; TRo/CSm: the energy flux captured by the active reaction center of PSII per unit leaf section; φ_Do_: quantum ratio used for heat dissipation at t = 0; DIo/CSm: the energy of heat dissipation per unit leaf cross-section. The specific activity parameters accurately reflect the absorption, transformation, and dissipation of light energy by the photosynthetic organs when the fluorescence reaches its maximum. The different letters indicate significant differences as assessed by the Turkey HSD test (*p* < 0.05; *n* ≥ 4).

We also analyzed other PE-related parameters during recovery after CS-ACC. Compared with CK, F_V_/F_M_, F_V_/F_O,_ PI_ABS_, and ET_O_/CSm of cucumber seedlings decreased during recovery after CS-ACC except for F_V_/F_M,_ which showed no significant difference after 48 h of recovery (Figure 6A–D). ABS/CSm and TR_O_/CSm first decreased and then increased and even recovered to the CK level at 48 h of recovery (Figure 6E,F). Moreover, φ_Do_ and DI_O_/CSm first increased and then also recovered to the CK level at 48 h of recovery (Figure 6G,H). These results indicated that PE was gradually repaired during recovery after CS-ACC. However, inhibition of *RBOH*-dependent signaling resulted in lower F_V_/F_M_, F_V_/F_O,_ PI_ABS_, ET_O_/CSm, ABS/CSm, and TR_O_/CSm but φ_Do_ and DI_O_/CSm were higher during recovery after CS-ACC, indicating that PE of CS-ACC-DPI was suppressed.

**Figure 7:**
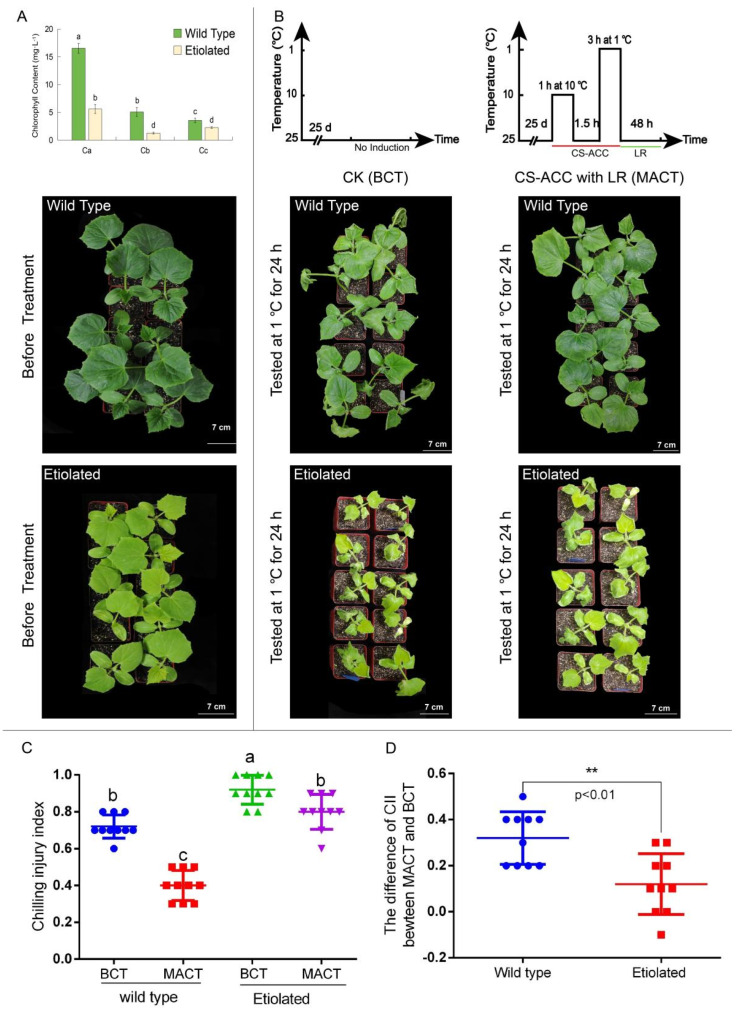
The photosynthetic efficiency was of vital importance to cold stress memory in cucumber seedlings. (**A**): the chlorophyll content and phenotype for the wild-type and etiolated cucumber seedlings; Ca: chlorophyll a; Cb: chlorophyll b; Cc: carotenoid. (**B**): the cold tolerance of the wild-type and etiolated cucumber seedlings after different treatments. CK: cucumber seedlings with no acclimation and at 25 °C during treatment; CS-ACC with LR: cucumber seedlings were treated with a cold stress regime of 10 °C for 1 h, 25 °C for 1.5 h, 1 °C for 3 h, and 25 °C for 48 h; CS-ACC: cold stress acclimation; LR: long recovery; BCT: basic cold stress tolerance; MACT: the maintenance of acquired cold tolerance. (**C**,**D**): CII of cucumber seedlings after different treatments. The different letters indicate significant differences as assessed by the Turkey HSD test (*p* < 0.05; *n* ≥ 9). ** represents the 0.01 level.

We further used etiolated cucumber seedlings to detect the importance of photosynthesis for cold stress memory (Figure 7). We found that the chlorophyll content of the etiolated seedlings was significantly lower than that of wild-type (WT) seedlings (Figure 7A), which indicated that the photosynthetic was lower than that of WT seedlings. The cold tolerance and CII of seedlings were tested by a tester cold stress (1 °C) following 48 h of recovery after different treatments. We found that CII of BCT and MACT for WT was significantly lower than that of etiolated seedlings (Figure 7B,C). Moreover, compared to the etiolated seedlings, the cold tolerance of WT was significantly improved after CS-ACC (Figure 7B,D), indicating that photosynthetic efficiency was of vital importance to cold stress memory in cucumber seedlings.

### 3.8. Genome-Wide Analysis of the BZR Family in Cucumber

The above promoter analysis showed that there are a large number of BZR transcription factor-binding activation sites in *RBOH* promoters. We further identified 4 *CsBZR* genes in the cucumber genome and named them *CsBZR1-4* according to their arrangement order on the chromosomes (Table S3). The 4 genes were distributed on chromosome 1, 2, 4, and 6 of cucumber, respectively (Figure S5). Subcellular localization prediction showed that these family members localized to the nucleus (Table S3).

We also constructed protein phylogenetic trees of the BZR family (Figure S6). The BZR rootless phylogenetic tree was divided into four groups. The groups 1 and 2 contained 25 BZR proteins, accounting for 86% of all the proteins examined in the phylogeny, while group 3 contained 1 protein and group 4 contained 3 proteins (Figure S6A). The 4 CsBZR proteins were distributed in groups 1 and 2, indicating that the specificity of CsBZR proteins in cucumber was higher and more evolved than that of the other plant species tested. Similarly, we investigated and identified 10 conserved motifs (Figures S6B and S7) and members of the same group retained similar motifs. Motif 1 had the largest width and highly conserved amino acid residues in the BZR domain. Groups 3 and 4 were only composed of motif 1 while similar motif compositions were retained in groups 1 and 2, including the 4 CsBZR proteins; however, *solyc12g089040.2.1* did not contain motif 1. These results also showed that besides *Solyc12g089040.2.1*, *OS02T0233200-00*, and *OS01T0176900-00*, the remaining *BZR* genes all contained two UTR and two CDS regions. Three *CsBZR* genes contained only one intron while *CsBZR3* had two introns (Figure S6C). Additionally, we also analyzed the gene linkage of the *CsBZR* family between cucumber and different plant species but found no collinearity.

### 3.9. Inhibition of BRs or RBOH-Dependent Signaling Affected the Maintenance of the Expression of Two CsBZRs during Recovery after CS-ACC

**Figure 8:**
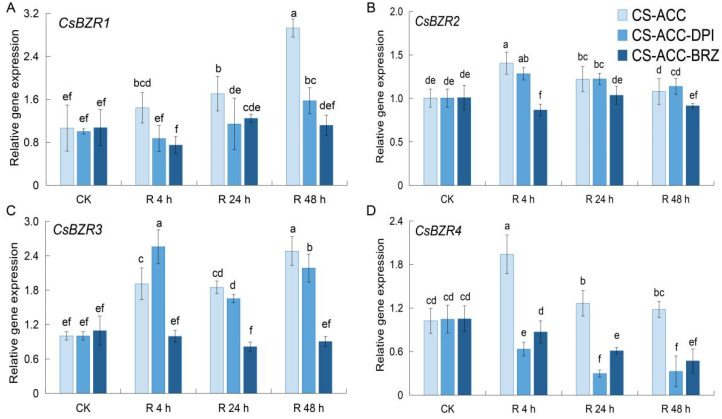
Inhibition of *RBOH*-dependent signaling or BRs during recovery after CS-ACC affected the expression of *CsBZR1* (**A**), *CsBZR2* (**B**), *CsBZR3* (**C**) and *CsBZR4* (**D**), respectively. CK: the control, cucumber seedlings with no acclimation and at 25 °C during recovery; CS-ACC: seedlings were treated with a cold stress regime of 10 °C for 1 h, 25 °C for 1.5 h, and 1 °C for 3 h; CS-ACC-DPI: DPI was sprayed onto the seedlings during recovery after CS-ACC; CS-ACC-BRZ: BRZ was sprayed onto the seedlings during recovery after CS-ACC; R: recovery, 25 °C. The different letters indicate significant differences as assessed by the Turkey HSD test (*p* < 0.05; *n* ≥ 4).

We further determined the expression pattern of the *CsBZR* family during recovery after CS-ACC. *CsBZR1* and *3* showed high expression during recovery after CS-ACC (Figure 8A,C). However, *CsBZR2* and *4* increased and then decreased during recovery after CS-ACC (Figure 8B,D). When DPI or BRZ were sprayed, *CsBZR*-CSM was decreased during recovery after CS-ACC except for *CsBRZ3* at 4 h of recovery after CS-ACC-DPI.

### 3.10. BRs Are Involved in the Cold Stress Memory of Cucumber Seedlings

**Figure 9:**
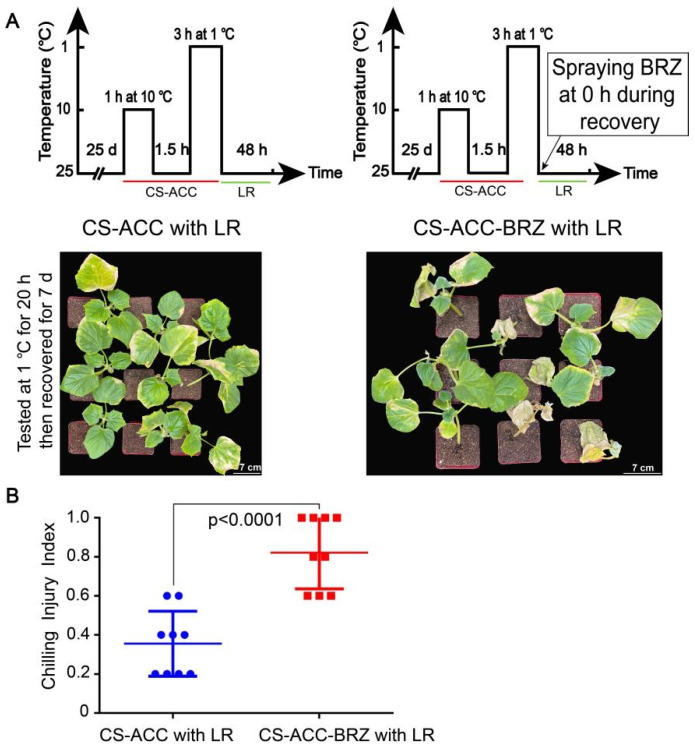
The cold tolerance (**A**) and CII (**B**) of cucumber seedlings tested by a tester cold stress (1 °C, 20 h) following 48 h of recovery after CS-ACC or CS-ACC-BRZ, and then placed in recovery for a period (25 °C/18 °C, day/night, 7 days). (**A**): a schematic diagram and cold tolerance of CS-ACC with LR and CS-ACC-BRZ with LR. CS-ACC: cucumber seedlings were treated with a cold stress regime of 10 °C for 1 h, 25 °C for 1.5 h, and 1 °C for 3 h; CS-ACC-BRZ: BRZ was sprayed onto the seedlings during recovery after CS-ACC. (**B**): CII of cucumber seedlings. CS-ACC: cold stress acclimation; LR: long recovery, 25 °C.

In the *CsBZR* family, we found that *CsBZR*-CSM genes showed high expression during recovery after CS-ACC. Therefore, we hypothesized that BRs may also participate in cold stress memory. The cold tolerance and CII of cucumber seedlings were tested by a tester cold stress (1 °C), following 48 h of recovery after CS-ACC or CS-ACC-BRZ. When the tester time reached 20 h, we found that after CS-ACC, CII was significantly lower than that of seedlings subjected to CS-ACC-BRZ (Figure 9). Thus, inhibition of BRs significantly reduced the cold tolerance of cucumber seedlings. The results indicated that BRs are also essential to the cold stress memory of cucumber seedlings.

## 4. Discussion

Plants experience abiotic stress due to continuous fluctuations in the external environment [1,69]. *Arabidopsis* experience abiotic stress due to drought [70], salt [71], temperature [13,69,72], and light intensity [73] conditions, among others. Moreover, the maintenance of acquired abiotic tolerance can enhance the tolerance of plants to various cross stresses in the dynamic environment. Currently, studies on the maintenance of acquired cold tolerance (MACT) have only been conducted in *Arabidopsis* and are limited to the mining of transcriptome and metabolome datasets [13]. However, the underlying mechanism of the signaling that regulates the MACT event in plants is still unclear.

### 4.1. Maintenance of Acquired Cold Tolerance Occurs in Cucumber Seedlings

Cold memory includes vernalization and cold stress memory. Studies have shown that vernalization and cold stress memory have different requirements and molecular mechanisms in response to low temperatures [3,74,75,76,77]. Cold stress memory results in MACT in plants [3,13]. The cold stress tolerance of plants can be divided into three types: basic cold stress tolerance (BCT), acquired cold stress tolerance (ACT), and MACT [3,78,79]. At present, most research has mainly focused on BCT and ACT [76,77,80,81] rather than MACT. In this study, a tester cold stress was applied to determine the cold tolerance of the treatments. Cold tolerance was significantly enhanced following 1.5 h of recovery after 10 °C induction. In contrast, cold tolerance was significantly decreased following a 48 h recovery period after induction at 10 °C. However, the cucumber seedlings showed stronger cold tolerance following 48 h of recovery after CS-ACC. These results strongly indicated that CS-ACC resulted in the acquisition of cold stress memory in cucumber seedlings and enhanced MACT.

### 4.2. Both BRs and RBOH-Dependent Signaling Are Essential for Cold Stress Memory in Cucumber Seedlings

Plants respond to cold stress by activating multiple hormone signaling pathways [40,43,82,83,84]. However, BRs produce complex signals in response to cold stress, and might play a more important role in the cold tolerance of plants. Our previous studies have shown that *RBOH*-dependent signaling is involved in the regulation of heat stress memory in tomato [33,34] and a large number of BZR-binding sites exist in the promoter of the RBOH family. Therefore, we hypothesized that BRs might be involved in cold stress memory. BRs are known as plant growth regulators that not only participate in the growth and development of plants [41,42,54] but also play an important role in the regulation of low-temperature stress [40,85,86,87]. When tomato plants were exposed to low-temperature stress, exogenous BRs (EBR) enhanced the activity of antioxidant enzymes by decreasing the damage of reactive oxygen species (ROS) to improve the plants’ survival rate [88]. Treatment with EBR can improve the antioxidant enzyme activity in cucumber seedlings under low-temperature stress and inhibit excessive production of ROS and malondialdehyde, and protect the photosynthetic mechanism. This promotes the growth of cucumber seedlings [89]. *Respiratory burst oxidase homologues*-dependent (*RBOH*-dependent) ROS also plays an important signaling role in basal cold stress tolerance in plants [90,91]. Both *RBOH*-dependent signaling and BRs were involved in the regulation of the cold stress response while the BR-induced stress response was related to an increase in H_2_O_2_ produced by NADPH oxidase activity regulated by *RBOH* [40,43]. Previous research found that low-temperature stress caused a BRs signaling cascade reaction in tomato, in which a key transcription factor BZR1 directly activated the transcription of *RBOH1* and stimulated the accumulation of apoplast H_2_O_2_, thus regulating the transcription of *CBFs* and photoprotection, and subsequent cold tolerance [62,92]. However, whether *RBOH*-dependent signaling and BRs participate in the regulation of cold stress memory and the regulation mechanism itself remain unknown.

Our previous studies have shown that *RBOH*-dependent signaling is involved in regulation of the maintenance of heat stress in tomato [33,34]. Therefore, we hypothesized that *RBOH*-dependent signaling also participates in MACT of cucumber seedlings. In this study, we found that the activity of NADPH oxidase encoded by the *CsRBOH* family and the total H_2_O_2_ content showed higher levels during recovery after CS-ACC; however, inhibition of *RBOH*-dependent signaling during recovery after CS-ACC resulted in a decrease in NADPH oxidase activity and the total H_2_O_2_ content and the loss of MACT, indicating that *RBOH*-dependent signaling was essential for the cold stress memory of cucumber seedlings.

Since the *RBOH*-dependent signaling was reactive oxygen species-related signaling, we further explored the electrolytic leakage and proline content. We found that both indexes were significantly increased during 4 h of recovery after CS-ACC, indicating that the cucumber seedlings suffered oxidative stress injury. Further inhibition of *RBOH*-dependent signaling significantly increased the electrolytic leakage and proline content, suggesting that a reduction in *RBOH*-dependent signaling resulted in cucumber seedlings suffering more serious cold stress injury and this signaling was essential for cold stress memory to maintain relatively lower oxidative injury after CS-ACC, contributing to the increase in future MACT.

We then identified and characterized nine *CsRBOH* family members within the cucumber genome for the first time. We found that only one pair of *CsRBOH5* and *CsRBOH6* in cucumber itself had collinearity and were distributed in the highly specific group of the phylogenetic tree, indicating that the RBOH protein sequence of different species was conserved; however, the nucleotide of the *CsRBOH* genes had high specificity. We also found that *CsRBOH6* had collinearity with *Solyc08g081690* in tomato and *AT1G64060* (*RBOHsF*) in *Arabidopsis*; both *Solyc08g081690* and *AT1G64060* are involved in abiotic stress [26,93,94,95]. Therefore, we hypothesized that *CsRBOH6* and *CsRBOH*5 are two key *CsRBOHs* involved in abiotic stress in cucumber. Further study found that *CsRBOH2*, *3*, *4*, and *5* showed high expression during recovery after CS-ACC and a large number of brassinosteroids (BRs) transcript factor-binding sites were identified in the promoter of these memory-responsive *CsRBOH* genes; however, inhibition of *RBOH*-dependent signaling or BRs affected the expression of these memory-responsive *CsRBOH* genes. Additionally, we also found that *CsBZR1* and *3* showed high expression during recovery after CS-ACC. Inhibition of *RBOH*-dependent signaling or BRs during recovery after CS-ACC also affected the expression of *CsBZR1* and *3* and resulted in the loss of MACT. Therefore, *RBOH*-dependent signaling and BRs are essential for the cold stress memory of cucumber seedlings.

### 4.3. The Repair of Photosynthetic Efficiency Mediated by CsRBOHs-Dependent Signaling during Recovery after CS-ACC Is Important for Cold Stress Memory in Cucumber

Under low temperatures, the increased expression of *RBOH* in cucumber seedlings can increase the accumulation of endogenous H_2_O_2_, promote CO_2_ assimilation, and induce PSI and PSII photoprotection to improve photosynthesis in seedlings [96]. However, it is still unclear whether *RBOH*-dependent signaling regulates the photosynthesis-related process during recovery after CS-ACC, affecting cold stress memory.

Chlorophyll fluorescence parameters have been widely used in the study of plant environmental stress [51,52]. It was found that the photosynthetic fluorescence parameter Fv/Fm is the key index for evaluating plants’ cold tolerance due to its quick response and significant variation in plants under cold stress [97,98,99,100]. In this study, we detected the changes in the photosynthetic efficiency (PE)-related fluorescence induction curve and parameters, including Fv/Fm, of cucumber seedlings during recovery after CS-ACC. Interesting, we found that the PE-related parameters during recovery after CS-ACC first decreased and then increased and recovered to the CK level at 48 h of recovery. Meanwhile, the fluorescence induction curve during recovery after CS-ACC was gradually lower than that of CK during recovery at 24 and 48 h after CS-ACC. These results revealed that PE was enhanced during recovery after CS-ACC, which suggests that the repair of PE during recovery after CS-ACC may benefit cold stress memory. Further inhibition of *RBOH*-dependent signaling significantly suppressed PE of cucumber seedlings during recovery after CS-ACC, resulting in the loss of cold stress memory and a decrease in MACT of cucumber seedlings, which may be due to the imbalance between photosynthetic repair and stress resistance. Furthermore, we assessed the importance of PE for cold stress memory using etiolated seedlings. The results showed that the cold tolerance of wild-type seedlings was significantly higher than that of etiolated seedlings after cold stress treatment. Compared to the etiolated seedlings, the cold tolerance of wild-type cucumber seedlings was significantly improved after CS-ACC, indicating that PE was of vital importance for the cold stress memory of cucumber seedlings. Another study found that when plants were subjected to extreme low temperatures, they maintained the fluidity of their membranes to survive this environmental stress, and the degree of the membranes’ fluidity further affected the absorption, transmission, and utilization of light energy by photosynthetic pigments [12,101,102]. Furthermore, the balance between photosynthetic repair energy and stress resistance energy is crucial for plants’ cold tolerance [44,45,46]. Therefore, we hypothesized that these factors are not incompatible, and the balance between the repair of plants’ PE and stress tolerance is important for cold stress memory in cucumber seedlings.

## 5. Conclusions

We found that (1) cold stress induced the acquisition of cold stress memory and enhanced the maintenance of acquired cold tolerance (MACT) in cucumber seedlings; (2) both *RBOH*-dependent signaling and BRs were essential for the cold stress memory of cucumber seedlings; and (3) *RBOH*-dependent signaling was essential for MACT as it altered the photosynthetic efficiency during recovery after CS-ACC in cucumber seedlings.

In general, this study provided new ideas for the study of abiotic stress resistance and its mechanism. More importantly, this study contributes to the improvement of plants’ cold tolerance, and also has important theoretical significance and scientific value for enriching the mechanisms of plants’ resistance to abiotic stress in natural environments. Next, it is necessary to further study the duration of cold stress memory and the acquisition cold tolerance intensity of cucumber in different genotypes to lay the foundation for future applications of overwinter cultivation of cucumber.

## Data Availability

All datasets generated for this study are included in the article or Supplementary File.

## Supplementary Materials

The following are available online at https://www.mdpi.com/article/10.3390/antiox11050969/s1, Figure S1: Distribution of nine *CsRBOH* genes in the cucumber chromosome; Figure S2: Bioinformatics analysis of the phylogenetic relationships, architecture of conserved motifs, and gene structure of the RBOH family in different plant species; Figure S3: Detail information of the 10 conserved motifs in RBOH proteins, which were predicted with the MEME Suite; Figure S4: Collinearity analysis of the RBOH family between different plant species; Figure S5: The relative position of four *CsBZR* genes in the cucumber chromosome; Figure S6: Bioinformatics analysis of the phylogenetic relationships, architecture of conserved motifs, and gene structure of the BZR family in different plant species; Figure S7: Detail information of the 10 conserved motifs of BZR proteins; Table S1: Specific primer sequences for *CsRBOH* and *CsBZR*; Table S2: Identification and characteristics of *CsRBOH* family in cucumber; Table S3: Identification and characterization of *CsBZR* gene family in cucumber.
