# Supplementary material for: Photosynthesis Mediated by RBOH-Dependent Signaling Is Essential for Cold Stress Memory
Source: Antioxidants (Basel). 2022 May 14;11(5):969. doi: 10.3390/antiox11050969 (PMC9137663; doi:10.3390/antiox11050969)
Supplement: Supplementary file 1 [file antioxidants-11-00969-s001.zip › antioxidants-1717991-supplementary.pdf]

Supplemental Material

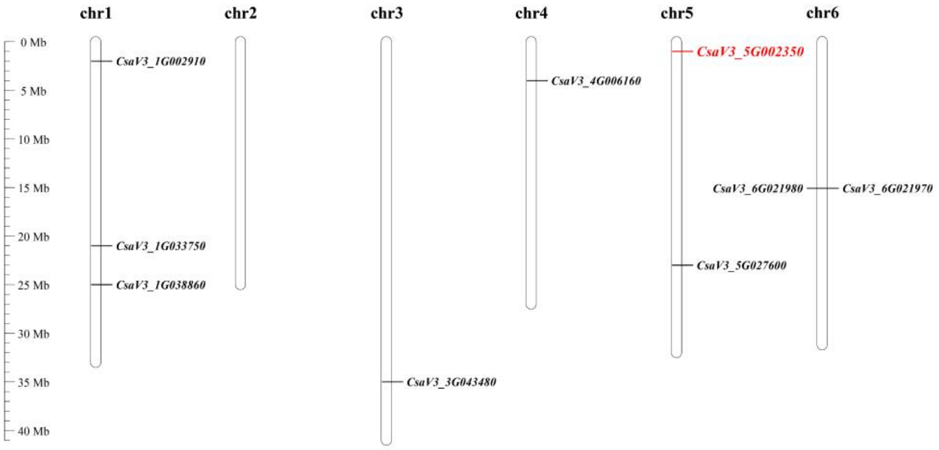

Figure S1. Distribution of nine *CsRBOH* genes in the cucumber chromosome. Table S2. Bioinformatics analysis of the phylogenetic relationships, architecture of conserved motifs, and gene structure of the RBOH family in different plant species. A: A total of 40 RBOH protein sequences were used to construct an rootless phylogenetic tree using MEGA7.0 software. The black squares represent the nine members of the *RBOH* family in cucumber. B: The motif domain analysis of different RBOH, where the numbers 1-10 in different colored boxes at the top-right represent different motifs. C: The untranslated 5′- and 3′- regions (green), CDS (yellow) and introns (black short lines) of the *RBOH*. The scale at the bottom of the figure estimates the length of the gene segment.

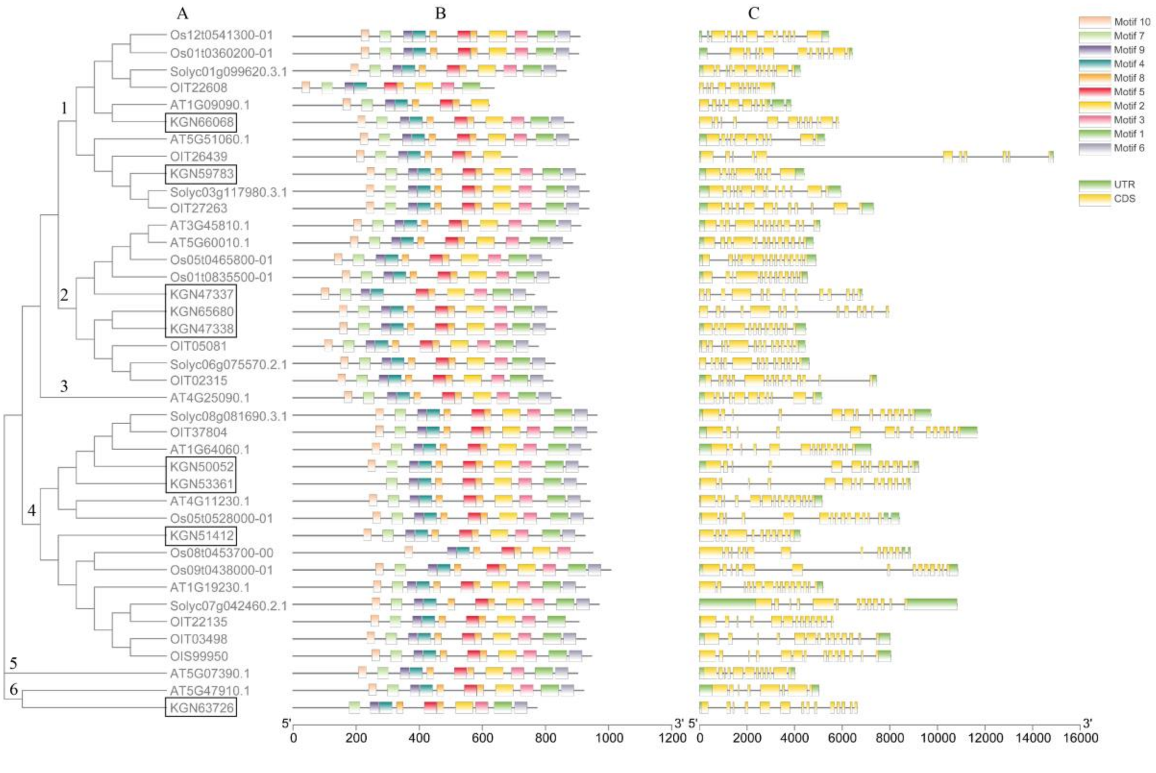

Figure S2. Bioinformatics analysis of the phylogenetic relationships, architecture of conserved motifs, and gene structure of the RBOH family in different plant species. A: A total of 40 RBOH protein sequences were used to construct an rootless phylogenetic tree using MEGA7.0 software. The black

squares represent the nine members of the RBOH family in cucumber. B: The motif domain analysis of different RBOH, where the numbers 1-10 in different colored boxes at the top-right represent different motifs. C: The untranslated 5′- and 3′- regions (green), CDS (yellow) and introns (black short lines) of the RBOH. The scale at the bottom of the figure estimates the length of the gene segment.

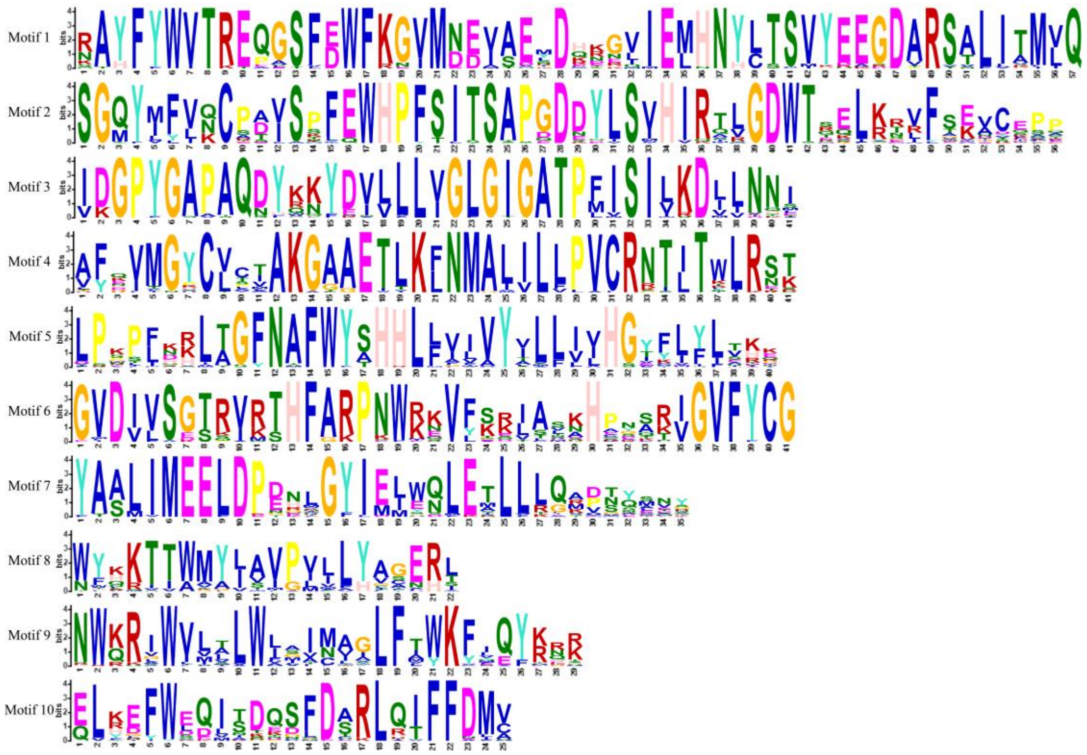

Figure S3. Detail information of the 10 conserved motifs in RBOH proteins, which were predicted with the MEME Suite. The relative sizes of letters indicate how often they occur in the sequence. Each position letters are arranged in descending order of the conservation. Different letter colors mean different amino acids.

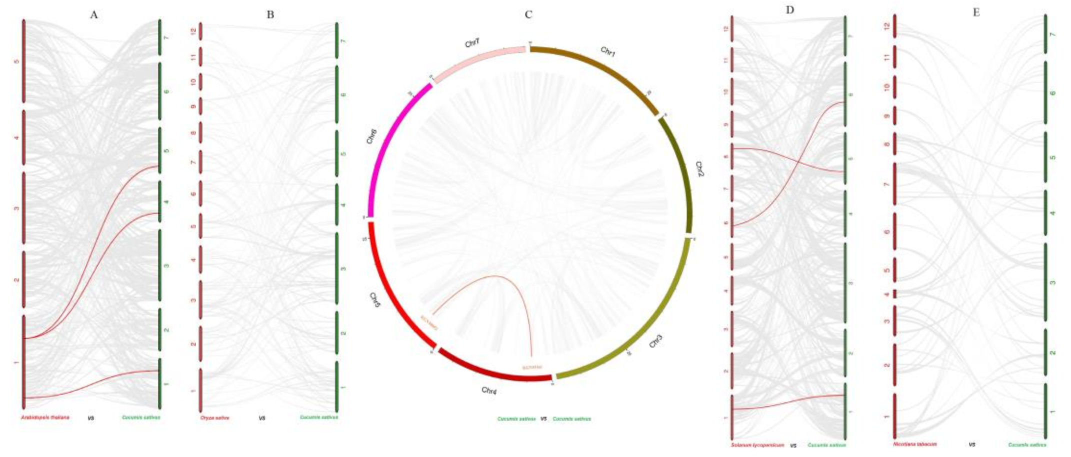

Figure S4. Collinearity analysis of the *RBOH* family between different plant species. Gray lines indicate the homology blocks between cucumber and different plants, while the red lines highlight the two linked genes that have direct homology. A, B, D, E: the collinearity analysis of cucumber and Arabidopsis, rice, tomato, and tobacco, respectively. C: the collinearity analysis of interchromosomal relationships within cucumber itself.

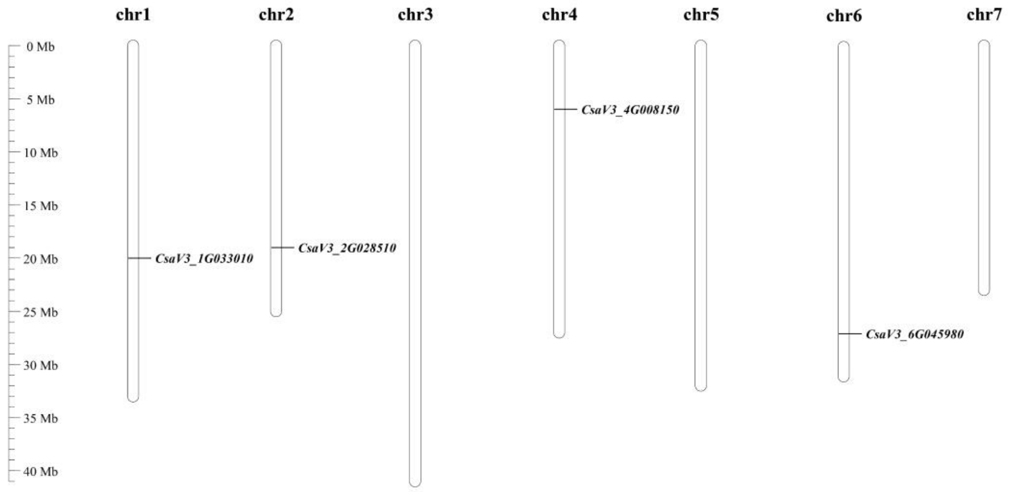

Figure S5. The relative position of four *CsBZR* genes in the cucumber chromosome. There are seven chromosomes that make up the cucumber genome. The ruler on the left represents the length of the chromosomes.

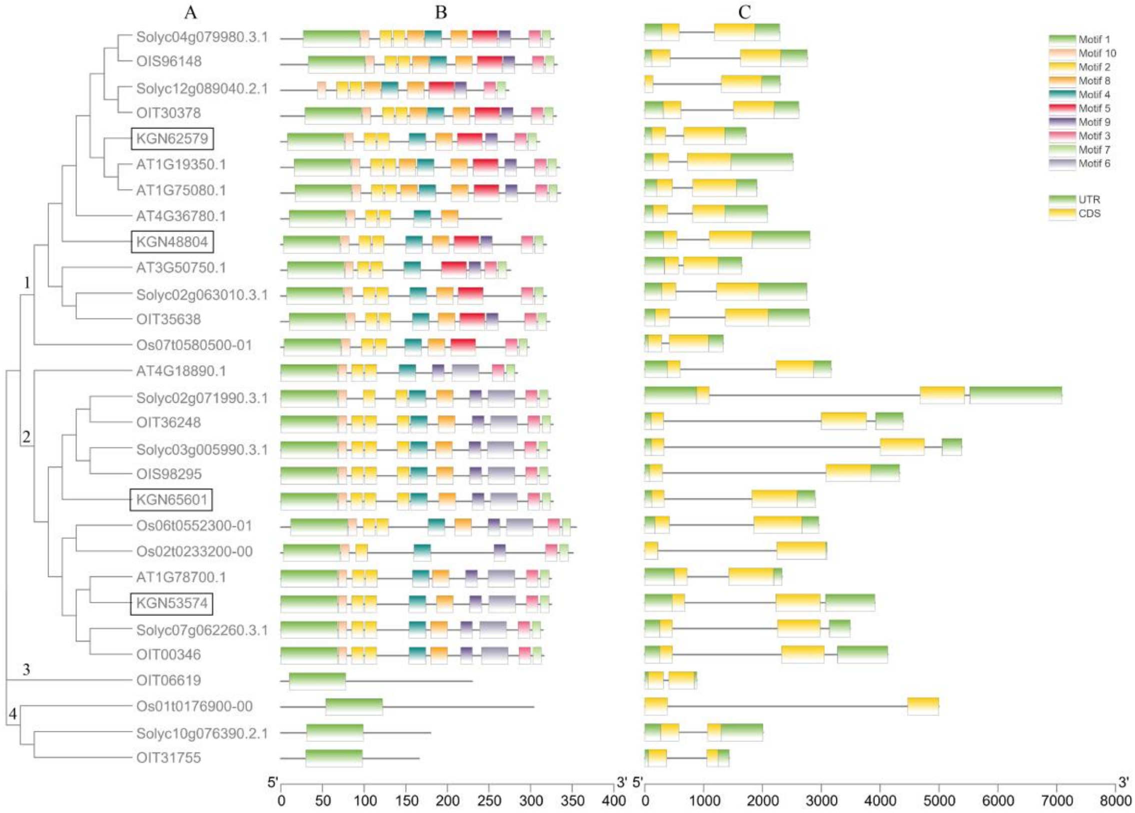

Figure S6. Bioinformatics analysis of the phylogenetic relationships, architecture of conserved motifs, and gene structure of the BZR family in different plant species. A: A total of 29 BZR protein sequences were used to construct an rootless phylogenetic tree. The black squares represent the four members of the *BZR* family in cucumber. B: The motif domain analysis of different BZR, where the numbers 1-10 in different colored boxes at the top-right represent different motifs. C: The untranslated 5′- and

3′- regions (green), CDS (yellow) and introns (black short lines) of the *BZR*. The scale at the bottom of the figure estimates the length of the gene segment.

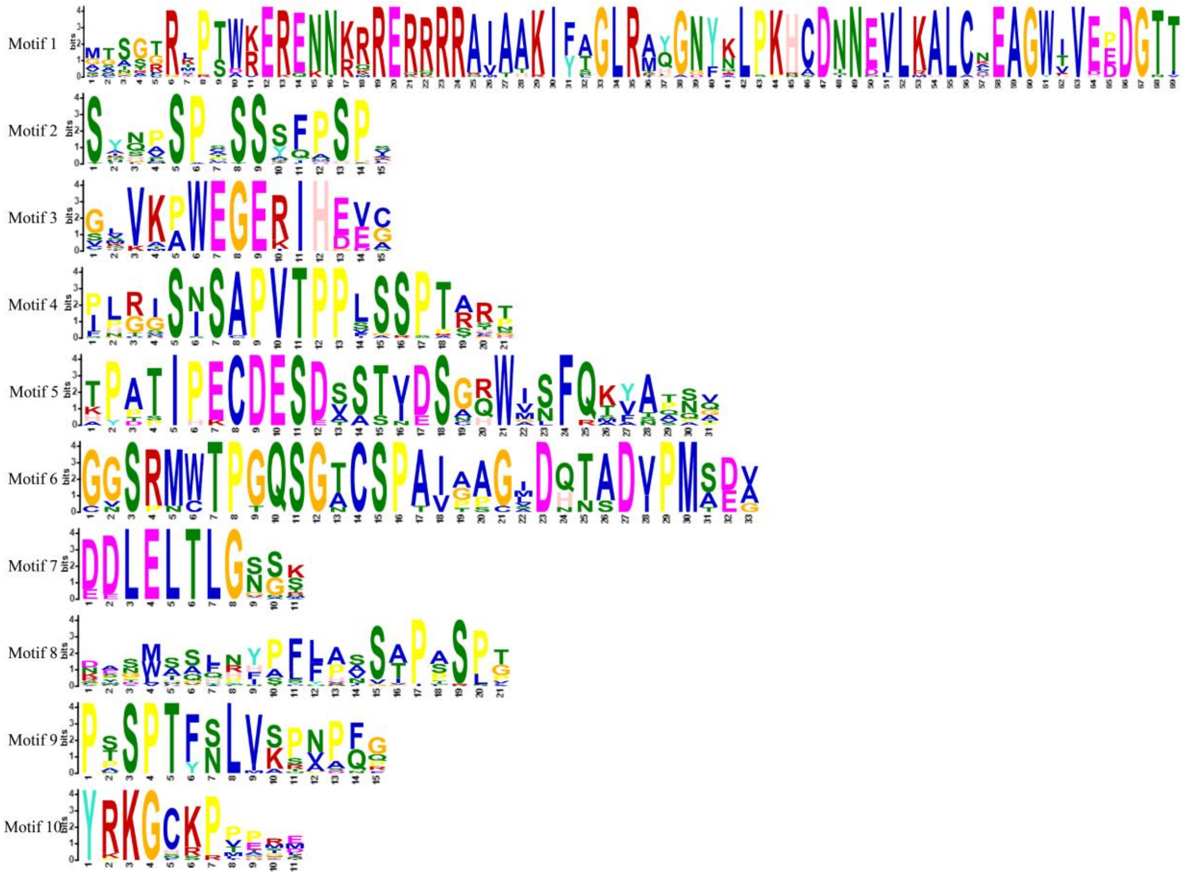

Figure S7. Detail information of the 10 conserved motifs of BZR proteins. The relative sizes of letters indicate how often they occur in the sequence. Each position letters are arranged in descending order of the conservation. Different letter colors mean different amino acids.

Table S1. Specific primer sequences for *CsRBOH* and *CsBZR*.

Gene Name	Gene ID (V3.0 version)	Forward Primer	Reverse Primer
CsTUA (Actin)	*CsaV3_4G000060*	ACGCTGTTGGTGGTGGTAC	GAGAGGGGTAAACAGTGAATC
CsRBOH1	*CsaV3_1G002910*	CCACGATTCATTTCAAGAGTCC	GATGATCAACCACAATGCTACC
CsRBOH2	*CsaV3_1G033750*	TCCAAAAAGATACCGAACTCCA	GTTGAATTTGGTTGTCTCAGCT
CsRBOH3	*CsaV3_1G038860*	TGCTGAAGAATATGCTGCTCTA	CAACAAAGTAGTGGAGTGAACG
CsRBOH4	*CsaV3_3G043480*	GCGTAACATTACTGGTGATTCC	ATTCTTCCATCAGCATCCGTAT
CsRBOH5	*CsaV3_4G006160*	ATCGGACGATATTACACAGCTT	GATCTAGAAGTCGAAGGGGAAG
CsRBOH6	*CsaV3_5G002350*	GCTATTGTAGTTGGCGTCATTC	CCATCCCTTAACCAAGTCTAGG
CsRBOH7	*CsaV3_5G027600*	CATTTACACTTGCTACTCGTCG	GTAATACTCTGATCTGCAGGCT
CsRBOH8	*CsaV3_6G021970*	AAAAATAAAGCCTGACGATGGG	CAAAGAAAATGGCAAGCCTAGT

CsRBOH9	*CsaV3_6G021980*	GTGCAATCTCCCATCAACTTTT	CACCACTCGTATTTCAAGCTTC
CsBZR1	*CsaV3_1G033010*	TTGGGAAGGAGAGAGAATTCAC	TTAAGAACTAGCAACTCCCGTT
CsBZR2	*CsaV3_2G028510*	GTTACGATGCCAATAATCCGTC	GATTCTAAGAGGAGGAAGCGAA
CsBZR3	*CsaV3_4G008150*	ATGGAAGTTCACTTATCCCCTG	ATGGCTGGGAGGAATAGTATTG
CsBZR4	*CsaV3_6G045980*	CTCTCTCTCTTTCCCAATCGAA	CGATTCTTCAGTTCCGACAATC

Table S2. Identification and characteristics of *CsRBOH* family in cucumber.

Gene name	Gene ID (V3.0 version)	Gene strand	Gene locus/bp	Gene length/bp	Protein ID	Gene chromosome	Subcellular Location
CsRBOH1	*CsaV3_1G002910*	-	1817861-1824687	6827	KGN63726	chr1	cell membrane
CsRBOH2	*CsaV3_1G033750*	-	20850248-20858115	7868	KGN65680	chr1	cell membrane
CsRBOH3	*CsaV3_1G038860*	-	24470328-24477603	7276	KGN66068	chr1	cell membrane
CsRBOH4	*CsaV3_3G043480*	+	35321657-35326379	4723	KGN59783	chr3	cell membrane
CsRBOH5	*CsaV3_4G006160*	+	4078163-4088463	10301	KGN53361	chr4	cell membrane
CsRBOH6	*CsaV3_5G002350*	-	1426967-1436539	9573	KGN50052	chr5	cell membrane
CsRBOH7	*CsaV3_5G027600*	-	22576940-22581504	4565	KGN51412	chr5	cell membrane
CsRBOH8	*CsaV3_6G021970*	-	14962962-14969982	7021	KGN47337	chr6	cell membrane
CsRBOH9	*CsaV3_6G021980*	-	14974893-14979791	4899	KGN47338	chr6	cell membrane

Table S3. Identification and characterization of *CsBZR* gene family in cucumber.

Gene name	Gene ID (V3.0 version)	Gene strand	Gene locus/bp	Gene length/bp	Protein ID	Gene chromosome	Subcellular Location
CsBZR1	*CsaV3_1G033010*	-	20054584-20057911	3328	KGN65601	chr1	nuclear
CsBZR2	*CsaV3_2G028510*	+	18745171-18747558	2388	KGN62579	chr2	nuclear
CsBZR3	*CsaV3_4G008150*	+	5720744-5727131	6388	KGN53574	chr4	nuclear
CsBZR4	*CsaV3_6G045980*	+	27211527-27214310	2784	KGN48804	chr6	nuclear
